# Incentives for Danish healthcare management based on a pilot outcome-based, patient-centric management model in psoriasis and psoriatic arthritis: the non-interventional IMPROVE study

**DOI:** 10.1186/s13690-020-00479-y

**Published:** 2020-10-12

**Authors:** Simon Francis Thomsen, Lone Skov, Lars Erik Kristensen, Morten Størling Hedegaard, Jakob Kjellberg, Tanja Schjødt Jørgensen, Søren Brenøe, Rikke Dodge

**Affiliations:** 1 Department of Dermatology, Bispebjerg Hospital & Department of Biomedical Sciences, University of Copenhagen, Copenhagen, Denmark; 2 Department of Dermatology and Allergy, Herlev and Gentofte Hospital, University of Copenhagen, Hellerup, Denmark; 3 The Parker Institute, Copenhagen University Hospital, Bispebjerg and Frederiksberg, Frederiksberg, Denmark; 4 Novartis Healthcare A/S, Copenhagen, Denmark; 5 KORA, Copenhagen, Denmark; 6 Copenhagen Economics, Copenhagen, Denmark

**Keywords:** Psoriasis, Psoriatic arthritis, Patient-centered healthcare, Value-based care

## Abstract

**Background:**

Psoriasis (PsO) and psoriatic arthritis (PsA) are chronic diseases that affect patients’ quality of life. The purpose of the present study was to develop a pilot outcome-based, patient-centric management model for PsO and PsA.

**Methods:**

The non-interventional IMPROVE (Incentives for healthcare management based on patient-related outcomes and value) study being conducted in Denmark consists of 5 phases: 1) collecting real-world evidence to estimate treatment patterns and disease burden to the healthcare sector and patients; 2) identifying disease aspects which matter most to patients by use of concept mapping; 3) conducting interviews with healthcare professionals and patient organization involved in a typical PsO or PsA patient journey in order to determine relevant measures to quantify patient-identified outcomes; 4) developing a value-based remuneration model based on outcomes from phases 1–3; and 5) testing the outcome-based model in pre-selected hospitals in Denmark.

**Results:**

Both PsO and PsA are associated with multiple co-morbidities, increased healthcare costs, and loss of earnings. Seven important ‘clusters’ of disease aspects were identified for both PsO and PsA, including uncertainty about disease progression and treatments, as well as inter-personal relations with healthcare providers. Hospital-based treatment was associated with high treatment costs. Although the outcome-based model could result in strategic behavior by doctors, those involved in defining the best outcome goals consider it unlikely.

**Conclusion:**

The new patient-centric outcome-based management model is expected to support optimal treatment and secure best possible outcomes for patients suffering from PsO or PsA. The practical implication of the present study are that the models developed are expected to increase focus on patient-centered healthcare, and help eliminate some of the inappropriate incentives that exist in activity-based remuneration systems.

**Trial registration:**

Not applicable; data collected from patient registries in Denmark.

## Background

Psoriasis (PsO) is a chronic, inflammatory, highly visible skin disease. Psoriatic arthritis (PsA) is a chronic inflammatory disease associated with irreversible bone and joint damage which can cause severe pain and restrict movement. In Europe, the prevalence of PsO is reported to range between 2 and 3%, and of PsA to range between 0.3–0.5% [1, 2]. In Denmark, the prevalence of PsO was estimated to be approximately 2.2%, and the number of adult patients with PsO was estimated to be approximately 126,055 [3].

Patients with PsO are at an increased risk of developing several comorbidities, including PsA [4–12]. Up to 30% of patients with PsO develop PsA over time; however, PsA can also be present in the absence of PsO [13]. Presence of PsA increases risk of cardiovascular (CV) diseases, Type II diabetes, hypertension, and other comorbidities [14–18]. Both PsO and PsA impact patients’ Quality of Life (QoL), resulting in a significant burden on the healthcare system, the economy, and society as a whole [19, 20].

There is pertinent proof that psoriasis has a negative effect on patient functionality and overall wellbeing. Although it is one of the most widely used measures in psoriasis research, the Psoriasis Area and Severity Index (PASI) is only modestly correlated with patient-reported outcome (PRO) measures of psoriasis and it may not reflect the most important aspects of psoriasis for the patient. Patients, however, state that these tools do not provide a holistic view of the extent to which psoriasis affects their life [21] .Currently, there are clinician-reported instruments for these two diseases that provide valuable information on disease severity and outcomes, and questionnaires, such as the Dermatology Life Quality Index (DLQI) for PsO, and the Health Assessment Questionnaire-Disability Index (HAQ-DI) for PsA, which measure the impact of disease on patients’ QoL. To improve outcomes for patients with PsO and PsA, the healthcare sector, and society in general, there is a need for an outcome-based, rather than an activity-based, healthcare management system. There is need for a holistic disease-management model to quantify the social consequences related to these diseases.

The aim of the IMPROVE (Incentives for healthcare management based on patient-related outcomes and value) study was to develop and test a pilot outcome-based, patient-centric management model for patients suffering from PsO or PsA.

## Methods

The IMPROVE study is a non-interventional study being conducted in Denmark. It consists of the following five phases:

### Phase 1: Estimate current treatment patterns and disease burden to the healthcare sector and patients in Denmark

In this phase, real-world evidence was collected retrospectively to fully understand how patients with PsO or PsA were currently being treated in the current Danish healthcare system. The data sources used for this phase are shown in Table 1.

**Table 1:** The interview guide from Copenhagen Economics

Interview Guide	Questions asked
Interview person	• What is your background? • What is your role in relation to the treatment of psoriasis patients? • What motivates you to make an extra effort in your daily work with psoriasis patients?
The current psoriasis treatment	• Can you describe the treatment journey for a concrete psoriasis patient? • How much variability is there in the state of disease between different observations of the same psoriasis patients? • Which value do the different treatment options provide for psoriasis patients? • If you had the same budget, with otherwise entirely free hands, would you organize treatment different from today?
The economic management model	• What kind of economic management are you subject to today as a practitioner working with psoriasis patients? • How do you experience the incentives you are faced with? • Do you have suggestions for changes in the economic management you encounter in your work?
Patients results	• Who has influence on the results of treatment? • Which role does the patient’s behavior play for a treatment’s result(s)? • To what extent do random events have an impact on the results?
Conclusion	• Is there anything you would like to add or that we have not asked you about? • Thank you very much for your participation!

The objectives of this phase were to collect data on:

a) Incidence, prevalence, and onset of disease stratified by socioeconomic factors and geography (PsO and PsA); b) Correlation of comorbidities with PsO and PsA and their effect on the onset of disease; c) Treatments and patient journey, time from symptom onset to diagnosis, time from diagnosis to treatment (topical/systemic/biologic), effect of treatment on development of comorbidities, distribution of treatment, and treatments depending on socioeconomics and geography; d) Cost of illness for society, the healthcare system, and the patient: Diagnosis-related Group model (DRG) and Danish for Ambulatory patients Grouping System (DAGS) tariffs, resource use, effect of disease on patients’ labor market participation and effect of treatment on cost of illness.

Data sources included the Danish National Patient Register (NPR), The National Registries of Medicinal Product Statistics (RMPS), the Danish adverse drug reaction (ADR) Database, cause of death registry, and socio-economic status from the Danish Civil Registration System database. Patients included in the retrospective analysis had a hospital diagnosis of PsO and/or PsA and were compared with a control group, and matched (1:2) on age, gender, marital status, and municipality. PsO and PsA patient analysis was split into 4 study groups: 1) Hospital diagnosed PsO (L40.0: plaque PsO; L40.4 guttate PsO (The International Classification of Diseases [ICD]-10 coding); 2) Hospital diagnosed PsA (L40.5, L40.5, M07.3, M07.0, M07.1, M07.2 M45 [inclusive of all sub codes]); 3) Anatomical Therapeutic Chemical Classification (ATC) codes; 4) Only ATC-codes no diagnosis 5 years before and after. Control groups were matched 1:2 on age, gender, married/co-living and municipality. Patients were followed from 1998 to 2014, and analyses were made from the year of diagnosis ±15 years. The populations were drawn at their first contact in the NPR-register and the index date was the start date.

For cost analysis, eligible patients must have been alive for one whole year after the index date and the following after and before periods. A patient/control may not survive during a period, but they will not be excluded even though they do not have a full 1-year period alive, as long as they were alive at the beginning of the period. The DRG cost was allocated on the index date. Income analysis covered the period 1998–2013 (there is no income information for 2014). Income was calculated for the calendar year. Income was set to missing (not 0) for patients aged 0–15 years, and for those with income over 270,000 €/year.

Co-morbidity was pooled on the 22 World Health Organization (WHO)-chapters [22]. All diagnoses (main, action, and secondary) in the 3 years before and after the index date were included. As a result, only index dates in the period 2001–2011 were included for this analysis. A conditional logistic regression model was applied to estimate the difference between cases and controls for each of the 22 WHO-chapters. No formal power calculations were performed. KORA, the Danish Institute for Local and Regional Government Research, performed management analyses and quality control of phase 1 data.

The detailed phase 1 methodology is presented elsewhere [23].

**Table:** 

Data sources included:	
Danish National Patient Register (NPR),	
The National Registries of Medicinal Product Statistics (RMPS),	
The Danish adverse drug reaction (ADR) Database	
The International Classification of Diseases [ICD]-10	
Anatomical Therapeutic Chemical Classification (ATC) codes	
World Health Organization (WHO)-chapters	

### Phase 2: Identify which disease aspects matter most to patients

Phase 2 was concept mapping [24, 25], which is a formal group process with a structured approach to identify ideas on a specific topic of interest and to organize those ideas accordingly into cogent domains. The objective of phase 2 was to identify PsO and PsA patient-relevant measurements.

This phase was conducted at the Parker Institute, Frederiksberg Hospital, Copenhagen, Denmark with patients referred from the department of Rheumatology, Copenhagen University Hospital at Frederiksberg, Denmark, the Department of Rheumatology, Gentofte Hospital, Denmark and the Department of Dermatology, Bispebjerg Hospital, Denmark. Adults with a clinical diagnosis of PsA (ClASsification for Psoriatic ARthritis -CASPAR criteria [2] or PsO [*n* = 12]) confirmed by trained specialists from these hospitals were recruited. All patients provided written informed consent. Patients were asked to provide responses to an initial task: ‘Thinking as broadly as you can, please list all the things that concern you and/or are important to you in your everyday life in relation to your PsA or PsO.’

Overall, four patient focus groups (two with six PsO patients each and the other two with six PsA patients each) were conducted. Homogenous response, also known as ‘saturation,’ was achieved; this indicated that the number of patients included was sufficient and so the option to expand the number of concept mapping (CM) focus groups was not used. The focus groups consisted of the following activities:

a) Individual brainstorming on the initial task, generating statements; b) In a nominal group process, sharing statements with the group and clarifying the meaning, if necessary; c) Sorting of all statements by each patient in any way that made sense to them; d) Cluster analysis and multi-dimensional scaling of the sorted statements by use of specialized software (Concept Systems) [21, 26]; e) Creation of a concept map of the statements, organized into clusters, presented and discussed with patients; and, f) Revision of the concept map by participants, including labeling of each cluster, drawing associations and causal relations between clusters and identification of sub- or super-clusters.

All the participants involved in the focus group were asked to rate the importance of each statement on a five-point scale: 1) very important for PsA or PsO, 2) important for PsA or PsO, 3) moderate importance for PsA or PsO, 4) minor importance for PsA or PsO, 5) not important for PsA or PsO. The endpoints could be clinician- or patient-reported, such as standardized disease severity indicators based on the PASI (Psoriasis Area and Severity Index) score model combined with, for example, a patient-reported outcome, such as the Psoriasis Symptom Diary (PSD) [27], to fully capture the value for patients and society. Management, analysis, and quality control of phase 2 data, was carried out by the Parker Institute, part of Copenhagen University Hospital at Bispebjerg and Frederiksberg.

Data from the concept maps was consolidated by removing identical statements using standardized content analysis [28]. The reduced statement pool was independently thematically analyzed, preserving fine distinctions in the wording across statements. The exact wording of the statements and cluster labels from the participants was kept, and sub-clusters were given labels derived from specific statements. The mean and median rating of statement relevance within each sub-cluster was calculated.

### Phase 3: Determine a relevant measure to quantify patient-identified outcomes

Phase 3 was based on interviews with different stakeholders such as healthcare professionals and patient organizations involved in a typical PsO and PsA patient journey. The objective was to understand the patient journey and identify PsO and PsA disease outcome measurements, based on results from a group of experts. The experts chosen based on their role in a PsO or PsA patients’ journey through the Danish health system were Professor Simon Francis Thomsen, treating dermatologist at Bispebjerg Hospital, Professor Lone Skov, treating dermatologist at Gentofte Hospital, Professor Robin Christensen, Lars Erik Kristensen, Henrik Rindel Gudbergsen, Lars Werner, and Jeppe H. Munck. No formal power calculations were performed. A certain set of questions were used to guide the interviews in a similar fashion (Table 1).

### Phase 4: Develop a management-model with financial incentives that support individualized treatments to optimize patient-specific questions

In phase 4, a value-based remuneration model for PsO and PsA was developed by an economics consulting group (Copenhagen Economics, Denmark), based on outcomes from phases 1–3. The model was developed taking into account established principles for efficient healthcare systems and was a proposal for remuneration of the Danish health service based on the outcomes delivered. In order to ensure that the patients’ other health conditions and QoL were taken into consideration; two potential prediction models were identified. The prediction models were based on the PASI score, which is the most widely used score for assessing the effectiveness of PsO treatment and patient-reported outcome goals in clinical trials [29].

### Phase 5: Testing the outcome-based model in selected hospital(s)

In phase 5, the chosen outcome-based model is to be tested in the dermatology departments of Bispebjerg and Gentofte Hospitals to assess whether the model’s good characteristics in a theoretical context are also present in practice. Any deviations from the expected behavior would then be used to adapt and improve the model’s specifications and parameters.

Here, we report the results from phases 1–4 of the IMPROVE study.

## Results

### Phase 1

In total, 13,025 patients with PsO and 10,525 patients with PsA were identified from the patient registries in Denmark. Both PsO and PsA were associated with increased healthcare costs and loss of earnings for patients suffering from the disease [30] . There was a significant increase in the mean annual treatment costs post-diagnosis of PsO and PsA, and there were inequalities in income and employment rates compared with matched controls. A number of different comorbidities were associated with both PsO and PsA [23]. CV disease and associated risk factors were more prevalent in patients with PsO and PsA than in matched controls. Patients with PsO had a particularly increased risk of mortality and death at a younger mean age compared with those with PsA [23].

### Phase 2

Seven important ‘clusters’ of disease aspects were identified for PsO within the three superclusters of ‘Having psoriasis,’ ‘Treatment,’ and ‘Surroundings/treatment’ (Table 2). All clusters and sub-clusters within the supercluster of ‘Having psoriasis’ were considered important by patients (Table 2). Under the supercluster of treatment, patients considered biological therapy to be “miraculous”, and were “concerned” about what would happen if the medication stops working or if they are not given the medication anymore (Table 2).

**Table 2:** Important disease aspects for patients with PsO

Supercluster	Cluster	Subcluster	Representative statement	Cluster mean	Subcluster mean
Having PsO	Social and mental problems, shame	Self-worth disappears	Felt like a leper	4.0	4.0
	Discomfort, pain, symptoms	I think it has been a serious handicap	It’s really awful for us	3.7	3.7
Treatment	At the doctor’s/the doctor knows my body	It’s a matter of being taken seriously as a complete person	It’s difficult to relate to the figures quoted by the doctor	3.7	4.0
		Long waiting time for treatment	At first I was sent from pillar to post – they had no idea what was wrong with me	3.7	3.9
		How do I know I’m getting the best treatment?	I feel that the doctors are experimenting on me	3.7	3.9
		I don’t have any information - I’m worried	I’ve read that inadequate treatment can result in complications	3.7	3.5
	Medication and treatment	Biological therapy is miraculous!	All my symptoms disappeared in 14 days (using biological drugs)	3.5	4.9
		What happens if the medication stops working - or if I’m not given it anymore?	Will my symptoms come back? (major concern)	3.5	4.7
		I’m a bit cautious about what I put in my body (medicines)	I don’t want to take MTX due to the side effects - the package insert didn’t say what was good about MTX	3.5	3.9
		I have to take medication for the rest of my life	This isn’t the end (if this treatment doesn’t work, then it’s on to the next one)	3.5	3.2
		I’m getting good treatment	I think the hospital I attend is fantastic	3.5	3.2
		Side effects	My skin has got thinner because they’ve rubbed all kinds of creams into it	3.5	3.0
Surroundings	Relationships with partner/others	I feel very alone with my condition	I don’t understand why there aren’t network groups (the PsO association)	3.2	3.9
		Intimacy - what does my partner think?	What does my partner think about how I look? (‘my backside could give a baboon some competition’)	3.2	3.4
		What do other people think - do they find it repulsive?	I’ve met people who didn’t want to shake hands with me	3.2	3.0
		No understanding of the restrictions caused by my condition (I can’t have a dram)	A lot of people have useful advice for me – knowing better than I do	3.2	2.6
	Own attitude/personal view	My condition controlled my life until I learned to accept it	You have to make sure that it doesn’t take over	3.2	3.8
		Will my children also get it?/I don’t want to have children!	I decided early on that I did not want to bring children into the world – this was not going to happen to them	3.2	3.3
		I’ve decided that what other people think isn’t my problem	Getting to the point where you’re not afraid to be seen in public is a victory	3.2	3.2
		You learn to live with it	The condition is incurable	3.2	2.9
	Consequences of the condition/The condition itself	Consequences for working life	My condition rules me out for some jobs	3.2	3.7
		The importance of lifestyle	Stress can provoke the symptoms	3.2	3.1
		I’m restricted by symptoms and treatment	When planning a holiday, you have to allow for when medication has to be taken etc.	3.2	2.9

*MTX* Methotrexate, *PsO* Psoriasis

Similarly, for PsA, seven important clusters of disease aspects were identified within the three superclusters of ‘Living with the condition,’ ‘Treatment,’ and ‘Surroundings/treatment’ (Table 3). Not having information about what they can do for themselves, pain, psychological disturbance, worries about treatment and side-effects, and the feeling that there was no understanding for/faith in them were the most important sub-clusters of disease aspects for patients with PsA (Table 3).

**Table 3:** Important disease aspects for patients with PsA

Supercluster	Cluster	Subcluster	Representative statement	Cluster mean	Subcluster mean
Living with the condition	Worries about my condition/unanswered questions	I don’t have any information about what I can do for myself	What can I do? If I feel pain, should I stop doing it?		4.1
	Worries about my condition/unanswered questions	Is it getting worse? (I feel out of my depth)	How does the future look for me?		3.8
	Worries about my condition/unanswered questions	Unemployed – so what?	For how long can I continue to work?		3.2
	What you’re exposed to, feelings about it	Pain	I wake up during the night in severe pain		4.1
	What you’re exposed to, feelings about it	Mentally affected	Mentally affected – the condition gets me down		4.0
	What you’re exposed to, feelings about it	My body is curling up	Consequences of not being able to use my body – the underlying level is getting worse and worse		3.8
	What you’re exposed to, feelings about it	Tiredness	I have to sleep for a few hours when I get home from work		3.5
	What you’re exposed to, the feelings about it	Restrictions in daily life (frustrating)	Always dropping things – very frustrating		2.9
Treatment	Medication (effects and side effects)	Worries about the medication	If the medication doesn’t work, are things just going to be the same for the rest of my life?		4.0
	Medication (effects and side effects)	Side effects	I’m under a mental strain with the medication		4.0
	Medication (effects and side effects)	Can I go without the medication?	If my condition has got worse while I’ve been taking the medication – can I possibly go without it?		3.6
	Medication (effects and side effects)	Hopes for the treatment	The medication affects different people differently, and of course a lot of them have had only positive experiences		3.4
	Medication (effects and side effects)	Frustrated about the lack of effect	I’ve tried a lot of the biological drugs, but either they were ineffective or I could not tolerate them		3.3
	Own approach (I’m doing something myself)	I’m bearing up/finding a solution	You find that anything is possible		3.9
	Own approach (I’m doing something myself)	What I can do for myself?	I find out a lot about my condition before I see the doctor		3.4
Surroundings	Being treated as a patient (not a person, just part of the system)	I get the impression that the doctor has to ‘tick the boxes’	You can easily end up feeling like a laboratory animal		3.8
	Being treated as a patient (not a person, just part of the system)	It’s hard to have a say in the treatment	The doctor says that if I do not do as he tells me, he won’t refer me		3.8
	The encounter with the system (the municipality)	There is no understanding for/faith in me	The municipality does not believe what I say		4.5
	The encounter with the system (the municipality)	It’s very difficult to get help	Applying for a flexible-hours job was a struggle		3.0
	The encounter with the system (the municipality)	An exhausting struggle with the system	Contact with the public system is immensely exhausting		2.5
	It’s difficult having an invisible handicap	It’s hard for others to understand my condition	I find it difficult to admit that I can’t do the same things as before		3.6
	It’s difficult having an invisible handicap	I notice that my illness makes people anxious about contact	People are alarmed when you tell them you’re having chemotherapy (MTX)		3.3
	It’s difficult having an invisible handicap	It’s hard to explain the pain I’m in	I don’t feel that I’m taken seriously		3.2

### Phase 3

**Fig. 1:**
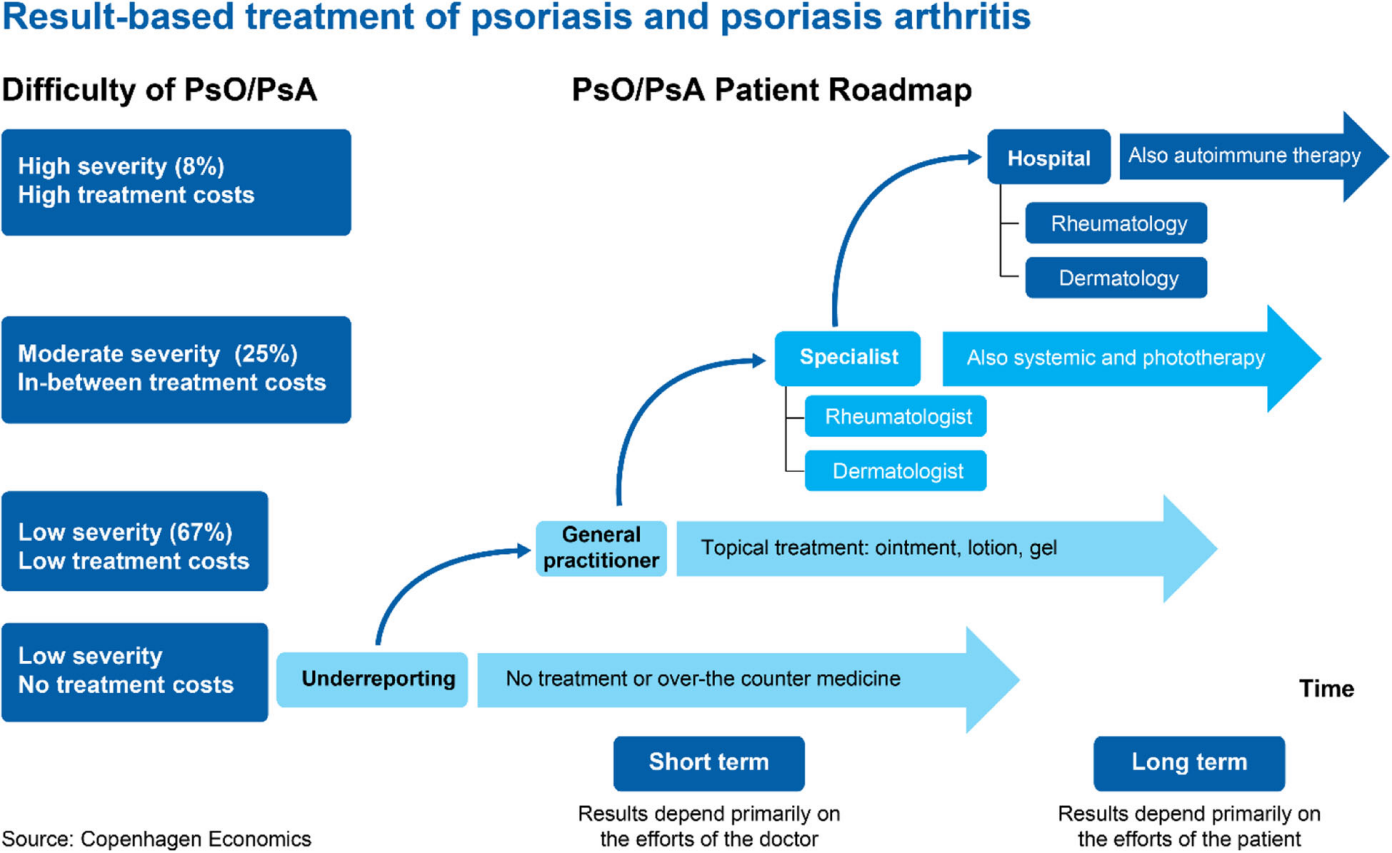
PsO/PsA Patient Roadmap. Dermatologist and Rheumatologist Interviews. PsA, psoriatic arthritis; PsO, psoriasis. The percentages describe the relative sizes of the patient population with high, moderate and low severity within the total population (100%)

Interviews with different healthcare professionals and patient organization showed that underreporting was common, and that hospital treatment was associated with high treatment costs. Topical treatment at the level of the general practitioner was considered easy and associated with low treatment costs (Fig. 1). The stakeholders’ response showed that while the short-term results of treatment depend primarily on doctor’s efforts, long-term results were dependent on those of the patient (Fig. 1).

**Fig. 2:**
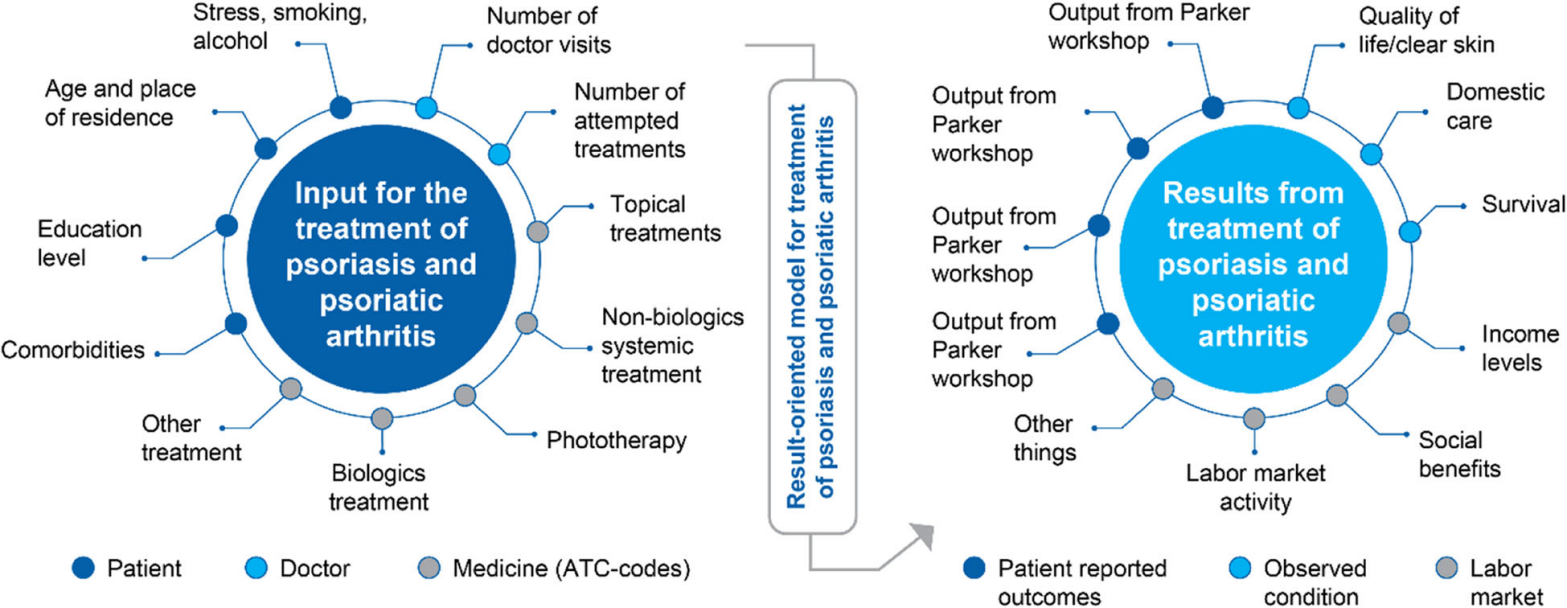
Input and outcomes from PsO and PsA treatment. ATC, anatomical therapeutic chemical classification code; PsA, psoriatic arthritis; PsO, psoriasis

For the prediction model for treatment of PsO and PsA, the inputs were obtained from patients on background factors such as comorbidities, education level, age, place of residence, stress, smoking, and alcohol consumption; and from doctors on number of visits, number of attempted treatments; and treatments used by ATC codes (Fig. 2). Treatment results were evaluated in terms of patient-reported outcomes (phase 2 results: based on importance and weightage for the different outcomes); observed conditions included QoL, income levels; and the labor market in terms of social benefit(s) and labor market activity (Fig. 2).

### Phase 4

The two prediction models developed (Model A and Model B) are described below.

In **Model A**, a prediction model was constructed using assessment (1), as shown below, in which the anticipated PASI after treatment (PS Exp.) was predicted based on the individual patient (‘p’s’) PASI before the treatment (PS Bef.) and based on obesity (K) and smoking (R) of KRAM (diet, smoking, alcohol, exercise) factors, comorbidities (KM) and other background variables, such as age and gender (BG). The β values are based on existing literature estimates [31–33].

1 $$ {PS}_{p, Exp.}={\beta}_0+{\beta}_1{PS}_{p, Bef.}+{\beta}_2\kern0.28em {KR}_p+{\beta}_3\kern0.28em {KM}_p+{\beta}_4\kern0.28em {BG}_p $$

Remuneration for the treatment of the individual patient ‘p’ was then determined based on the difference between the anticipated and the actual PASI (PS Act.) after treatment using assessment (2) shown below:

2 $$ A\;f\;l{.}_p=\left({PS}_{p, Exp.}-{PS}_{p, Act.}\right)\ast {\alpha}_p\ast k $$

In this equation, α was a factor that meant that the remuneration was corrected to reflect the extent to which the treatment takes account of the patients’ prioritization of a number of outcome goals as described in phase 3. If the treatment took into account those outcome goals that were important for the patient, the remuneration increased (α > 1), and if not, the factor fell (α < 1). Assessment (3) given below is an example of how α can be calculated based on two outcome goals:

3 $$ {\alpha}_p=\frac{0_1{w}_1+{0}_2{w}_2}{\left({0}_1+{0}_2\right)/2} $$

O was outcome goals and *ω* was the patient’s weighting of a given outcome goal. If the patient weighted all outcome goals equally or the treatment produced the same change in all outcome goals, there was no correction in α (α = 1) and the remuneration was based only on the PASI. *k* in assessment (2) above was a factor that determined the size of the reward for a more than expected reduction in the PASI. *k* was calibrated such that the maximum payment was at a level that was acceptable from a budgetary perspective. The final determination of α and *k* in assessment (2) were designed to ensure budgetary safety or to optimize the doctors’ incentive, irrespective of which was assigned the highest priority.

**Model B** was also based on literature estimates [31–33]. In this model, the placing in the spread of anticipated PASI after treatment was ascertained for each individual patient. The placing for an individual patient ‘ρ’ was calculated based on the deviation from the mean anticipated PASI expressed in standard deviations (SDs as shown in assessment (4) below:

4 $$ {Deviation}_p=\frac{PS_{p, Exp.}-{\mu}_{PS_{All, Exp.}}}{\sqrt{\frac{1}{n}{\sum}_p^n{\left({PS}_{p, Exp.}-{\mu}_{PS_{All, Exp.}}\right)}^2}} $$

where μ indicated the mean. The anticipated value of each outcome goal was calculated for the individual patient by assuming that they deviated from the mean in all patients in the same way for anticipated PASI. If the patient’s anticipated PASI was one SD less than the mean, the patient’s anticipated result was thus determined for all outcome goals as also being one SD less than the mean for the actual outcomes. The calculation for sub-outcome goal 1 was calculated as shown in assessment (5) below:

5 $$ {o}_{1,p, Exp.}={Deviation}_p\sqrt[\ast ]{\frac{1}{n}{\sum}_p^n{\left({o}_{1,p, Act.}-{\mu}_{o_{1, All, Act.}}\right)}^2}+{\mu}_{o_{1, Act.}} $$

**Fig. 3:**
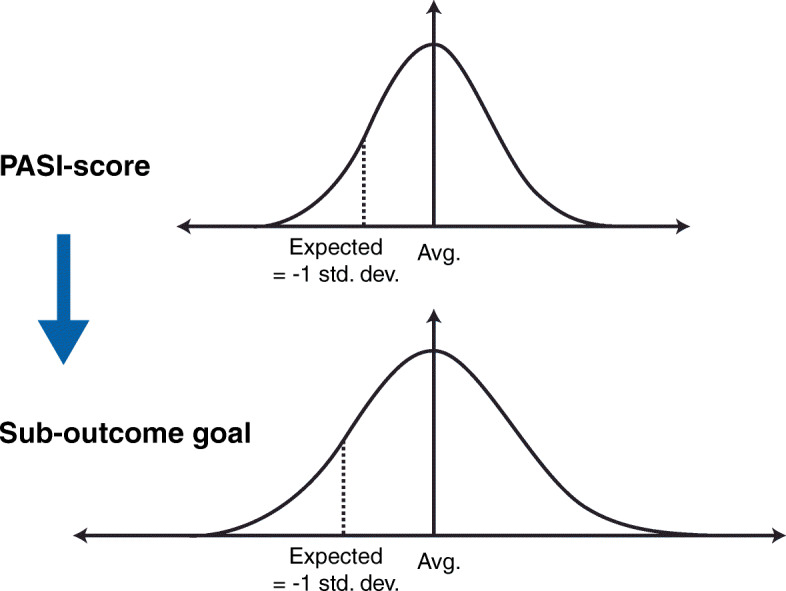
The assumption underlying assessment of calculation for sub-outcome goal 1. SD, standard deviation

The assumption underlying assessment (5) is illustrated in Fig. 3.

The remuneration for treatment of the individual patient ‘p’ was calculated as the change in the total weighted outcome goals consisting of i sub-goals as shown in assessment (6) below, where O_i_ was sub-outcome goal i and w_i_ was the individual patient’s weighting of the sub-outcome goal i.

6 $$ Paym{.}_p=\left(\sum \limits_{i=1}^I{w}_{i,p}\ast \left({o}_{i,p, Act.}-{o}_{i,p, Exp.}\right)\right)\ast k $$

k was, as in Model A, a factor that determined the size of the remuneration in order to reduce the PASI more than expected, and was calibrated such that the maximum payment was at a level that was acceptable from a budgetary perspective.

### Comparison of the two prediction models

The main difference between the two models was in whether the model rewarded an improvement in the PASI or an improvement in outcome goals. Model A rewarded improvements in PASI and corrected for whether the treatment took into account the patient’s wishes calculated by outcome goals. Model A was based on the doctor and patient working together to define the value of the treatment based on both clinical goals (PASI) and the patient’s prioritization of outcome goals. Model B rewarded improvements in outcome goals and used only the PASI to allow for the importance of KRAM factors, comorbidities, and background variables for ease of creating improvements in the outcome goal for the individual patient. Hence, Model B was based more on patients’ prioritization of outcome goals; relevant clinical goals were those which the patient found important in relation to living the life they want.

Once an actual and an anticipated outcome of the treatment were calculated for each sub-outcome goal, as described above, the total outcome of the treatment was calculated by weighting the individual sub-outcome goals with the weighting attributed to them by the individual patient, and then scaling them using a factor, k, as described in eq. (6) above. Establishing the factor k depended on which of the desired budgetary and incentive aspects were taken into account: 1) Compliance with an overall financial limit, and a limit to the reward, regardless of how large an improvement was created for the patient; 2) An incentive to achieve the best possible result for the individual patient, irrespective of the results for other patients; 3) Lack of possibilities to act strategically and exploit the remuneration system.

Because it may be difficult to satisfy all of these aspects at the same time, and in some cases, they may be mutually contradictory, the remuneration model ensured aspects 1 and 2 were met. With this model, although there is a theoretical risk of doctors acting strategically, it was believed that doctors’ focus on consideration for their patients will inhibit the risk of such behavior. This remuneration model proposes that the involved hospital department will receive the same amount of funds allotted under the de facto goal and limit control, but without the requirement for the now discontinued annual 2% improvement in productivity. An alternative to the 2% increase will, instead, be provided based on the patients’ outcome goals. This 2% of the hospital department’s budget then constitutes the financial limit which the value-based remuneration must adhere to. However, consideration for remuneration will only be given to those outcomes beyond the anticipated outcome, which are forecast in assessment (1) for Model A and assessment (5) for Model B.

### An example of the application of the remuneration model

For a given hospital department, if the overall improvement (which can be either PASI or outcome goals) for all patients was 10 units more than expected, and the result for the patients collectively was 3 units more than expected, then the department will receive 30% of the total limit as a reward for value. For Model B, factor k is calculated as in assessment (6), which determines remuneration of the individual hospital department as in assessment (7), where X is the total budget for value-based remuneration, n is all patients, and I is all sub-outcome goals.

7 $$ k=\frac{X}{\sum_p^n\left(\left({\sum}_{i=1}^I{w}_{i,p}\ast \left({o}_{i,p, Act.}-{o}_{i,p, Exp.}\right)\right)\right)} $$

This approach complies with aspect 1, as the amount paid out can never exceed the total limit X. At the same time, substantial consideration is given to aspect 2, as the individual hospital department will usually be able to achieve a greater proportion of that financial limit by creating an even better outcome for the patients. The only scenario in which this would not be the case is where a hospital department is the only one to create a better than expected outcome for the patients. In this case, this department would receive the full financial limit, regardless of how much better than expected the created outcome is.

In theory, this proposed approach does not conform to aspect 3 as described previously. This is because the doctors could, in principle, not agree to organize their treatment in order to create the greatest possible value for the patient. As long as the doctors create the same low value, they will achieve the same remuneration as they would achieve if they all created high value for their patients. This is the downside of making remuneration for the treatment of individual patients dependent on the total value created for all patients. It is, therefore, a ‘side effect’ of ensuring compliance with the overall budget. However, we do not expect this to be a major problem in practice, as strategic behavior by doctors is possible, albeit unlikely. Strategic behavior amongst doctors can be a serious concern only in cases doctors consider PASI or the defined outcome goals in Models A and B as irrelevant treatment goals. The doctors being involved in the work of defining the best outcome goals should counteract this.

## Discussion

In Denmark, there is an effort among leading decision makers in the healthcare system [34], including the Danish Regions [35] (the interest organization for the five Danish healthcare regions), the Ministry of Health, and the Ministry of Finance, to move from the current cost containment management model to a new outcome-based management model that remunerates the healthcare sector based on its ability to deliver on these outcomes. This study developed a new outcome-based, patient-centric management model for patients suffering from PsO and PsA but could also be applied to other disease areas.

Consistent with previous reports [19, 30, 36], in the first phase of this study, both PsO and PsA were found to be associated with increased healthcare costs and loss of earnings for patients suffering from the disease.

Concept mapping [24] used in the second phase of the study which focused on finding what patients regarded as important to them living a good life with their disease identified seven important ‘clusters’ of disease aspects for PsO or PsA; thereby providing new insights on patients’ perspectives on parameters that create true value for patients. Concept mapping is a highly effective method for the development of outcome measures [2, 37] to help guide the development of a value-based outcome model aimed at improving patient-HCP (healthcare professionals) interactions resulting in improved treatment management and optimal utilization of resources. In phase 3, interviews with different stakeholders involved in a typical PsO or PsA patient’s journey revealed underreporting of PsO and PsA, topical treatments being considered easy to use and of low cost, and hospital treatments being associated with high cost and more problematic. Patient-reported outcomes observed condition of the patient, and labor market were taken into consideration for evaluating treatment results.

Based on findings from phases 1–3, two value-based remuneration prediction models were developed in phase 4, and they differed in whether the incentives were for an improvement in the PASI or an improvement in outcome goals. The model based on improvement in outcome goals relied more on patient’s prioritization of outcome goals that defined the value of the treatment, and only those clinical goals were included which the patient found relevant in relation to living life the way they want. The potential downside of making remuneration for the treatment of individual patients dependent on the total value created for all patients is that of doctors creating the same low value, which would lead to achieving the same remuneration as they would have achieved if they all created high values for their patients. However, this is not expected to be a major problem in practice, as strategic behavior by doctors is unlikely, considering that doctors involved in defining the best outcome goals counteract the possibility. The next step would be to run a small pilot study in clinical settings to validate the model B for its proposed outcome goals.

The study had the following limitations: in phase 1, selection of groups 1 and 2 was based solely on diagnosis and treatment in a hospital setting, while groups 3 and 4 were selected based on prescription medication. In phase 2, the survey was unblinded and the survey design lacked a control group and did not assess differences between specific drugs or the impact of direct and indirect treatment costs. Phase 3 was based on personal interviews. Also, the study being conducted only in Denmark, limits the generalization of findings.

## Conclusions

Both PsO and PsA are associated with a substantial disease burden, including increased mortality risk and healthcare costs. The value-based control model developed in this study is expected to contribute to creating better outcomes for these patients by means of two primary channels. Firstly, once the new outcome-based, patient-centric management model is tested, it will support optimal treatment and secure best possible outcomes for patients suffering from PsO or PsA. Secondly, the model will eliminate some of the inappropriate incentives that exist in current activity-based remuneration systems, in which the health service is not incentivized for delivering better outcomes, if this occurs at a lower activity level. In the long term, the results from this study are expected to provide support to patients with PsA or PsO in improving self-management capabilities to motivate and empower them to take control of their treatment and strengthen implementation of treatment regimens for HCPs in clinical consultations.

## Data Availability

Data were collected from patient registries in Denmark, and in the present manuscript, we report 4 out of 5 phases of the study. Danish Institute for Local and Regional Government Research (KORA) helped with the register data, the Parker Institute with concept mapping and psoriatic arthritis expertise, and Copenhagen Economics with modelling.

## Abbreviations

ADR: The Danish adverse drug reaction Database
ATC: Anatomical Therapeutic Chemical Classification
BG: Background variables, such as age and gender
CASPAR: ClASsification for Psoriatic Arthritis
CV: Cardiovascular
DAGS: Danish for Ambulatory patients Grouping System
DLQI: Dermatology Life Quality Index
HAQ-DI: Health Assessment Questionnaire-Disability Index
DRG: Diagnosis-related Group model
HCP: Healthcare professionals
ICD: The International Classification of Diseases
IMPROVE: Incentives for healthcare management based on patient-related outcomes and value
KORA: The Danish Institute for Local and Regional Government Research
KRAM: Diet, smoking, alcohol, exercise
NPR: Danish National Patient Register
PASI: Psoriasis Area and Severity Index
‘p’s’: Individual patient
PsA: Psoriatic arthritis
PSD: Psoriasis Symptom Diary
PSForv: PASI after treatment
PSFakt.: difference between the anticipated and the actual PASI
PS Før: PASI before the treatment
PRO: Patient-Reported Outcome
PsO: Psoriasis
QoL: Quality of Life
RMPS: The National Registries of Medicinal Product Statistics
WHO: World Health Organization
SD: Standard Deviation

## References

[1] Springate DA, Parisi R, Kontopantelis E, reeves D, Griffiths CE, Ashcroft DM: incidence, prevalence and mortality of patients with psoriasis: a U.K. population-based cohort study. Br J Dermatol 2017, 176(3):650–658. doi:10.1111/bjd.15021. 10.1111/bjd.15021 PMC5363241 27579733

[2] Parisi R, Symmons DP, Griffiths CE, Ashcroft DM (2013). Global epidemiology of psoriasis: a systematic review of incidence and prevalence. J Invest Dermatol.

[3] Egeberg A, Skov L, Gislason GH, Thyssen JP, Mallbris L (2017). Incidence and prevalence of psoriasis in Denmark. Acta Derm Venereol.

[4] Yang YW, Keller JJ, Lin HC (2011). Medical comorbidity associated with psoriasis in adults: a population-based study. Br J Dermatol.

[5] Han C, Lofland JH, Zhao N, Schenkel B (2011). Increased prevalence of psychiatric disorders and health care-associated costs among patients with moderate-to-severe psoriasis. J Drugs Dermatol.

[6] Skolnick AH, Alexander ZJ (2006). Psychiatric implications of psoriasis. JAMA.

[7] Binus AM, Han J, Qamar AA, Mody EA, Holt EW, Qureshi AA (2012). Associated comorbidities in psoriasis and inflammatory bowel disease. J Eur Acad Dermatol Venereol.

[8] Gelfand JM, Neimann AL, Shin DB, Wang X, Margolis DJ, Troxel AB (2006). Risk of myocardial infarction in patients with psoriasis. Jama.

[9] Egeberg A, Mallbris L, Gislason GH, Skov L, Hansen PR (2016). Risk of multiple sclerosis in patients with psoriasis: a Danish Nationwide cohort study. J Invest Dermatol.

[10] Jensen P, Ahlehoff O, Egeberg A, Gislason G, Hansen PR, Skov L (2016). Psoriasis and new-onset depression: a Danish Nationwide cohort study. Acta Derm Venereol.

[11] Griffiths C, Jo S-J, Naldi L, Romiti R, Guevara-Sangines E, Howe T, Pietri G, Gilloteau I, Richardson C, Tian H, Augustin M (2018). A multidimensional assessment of the burden of psoriasis: results from a multinational dermatologist and patient survey. Br J Dermatol.

[12] Augustin M, Vietri J, Tian H, Gilloteau I (2017). Incremental burden of cardiovascular comorbidity and psoriatic arthritis among adults with moderate-to-severe psoriasis in five European countries. J Eur Acad Dermatol Venereol.

[13] Zachariae H, Zachariae R, Blomqvist K, Davidsson S, Molin L, Mork C, Sigurgeirsson B (2002). Quality of life and prevalence of arthritis reported by 5,795 members of the Nordic psoriasis associations. Data from the Nordic quality of life study. Acta Derm Venereol.

[14] Han C, Robinson DW, Hackett MV, Paramore LC, Fraeman KH, Bala MV (2006). Cardiovascular disease and risk factors in patients with rheumatoid arthritis, psoriatic arthritis, and ankylosing spondylitis. J Rheumatol.

[15] Khalid U, Ahlehoff O, Gislason GH, Kristensen SL, Skov L, Torp-Pedersen C, Hansen PR (2014). Psoriasis and risk of heart failure: a nationwide cohort study. Eur J Heart Fail.

[16] Khalid U, Hansen PR, Gislason GH, Lindhardsen J, Kristensen SL, Winther SA, Skov L, Torp-Pedersen C, Ahlehoff O (2013). Psoriasis and new-onset diabetes: a Danish nationwide cohort study. Diabetes Care.

[17] Ahlehoff O, Gislason GH, Lindhardsen J, Olesen JB, Charlot M, Skov L, Torp-Pedersen C, Hansen PR (2011). Prognosis following first-time myocardial infarction in patients with psoriasis: a Danish nationwide cohort study. J Intern Med.

[18] Setty AR, Curhan G, Choi HK (2007). Obesity, waist circumference, weight change, and the risk of psoriasis in women: Nurses’ health study II. Arch Intern Med.

[19] Feldman SR, Zhao Y, Shi L, Tran MH, Lu J (2015). Economic and comorbidity burden among moderate-to-severe psoriasis patients with comorbid psoriatic arthritis. Arthritis Care Res (Hoboken).

[20] Feldman SR, Tian H, Gilloteau I, Mollon P, Shu M (2017). Economic burden of comorbidities in psoriasis patients in the United States: results from a retrospective U.S. database. BMC Health Serv Res.

[21] Krueger G, Koo J, Lebwohl M, Menter A, Stern RS, Rolstad T (2001). The impact of psoriasis on quality of life: results of a 1998 National Psoriasis Foundation patient-membership survey. Arch Dermatol.

[22] World Health Organization. International statistical classification of diseases and related health problems, 10th revision, fifth edition, 2016: World Health Organization; 2015. https://apps.who.int/iris/handle/10665/246208.

[23] Skov L, Thomsen SF, Kristensen LE, Dodge R, Hedegaard MS, Kjellberg J (2019). Cause-specific mortality in patients with psoriasis and psoriatic arthritis. Br J Dermatol.

[24] Trochim W, Kane M (2005). Concept mapping: an introduction to structured conceptualization in health care. Int J Qual Health Care.

[25] Busija L, Buchbinder R, Osborne RH (2013). A grounded patient-centered approach generated the personal and societal burden of osteoarthritis model. J Clin Epidemiol.

[26] Heydendael VM, de Borgie CA, Spuls PI, Bossuyt PM, Bos JD, de Rie MA (2004). The burden of psoriasis is not determined by disease severity only. J Investig Dermatol Symp Proc.

[27] Lebwohl M, Swensen AR, Nyirady J, Kim E, Gwaltney CJ, Strober BE (2014). The psoriasis symptom diary: development and content validity of a novel patient-reported outcome instrument. Int J Dermatol.

[28] Crabtree B, Miller W, Crabtree BF, Miller WL (1999). Using Codes and Code Manuals: A Template Organizing Style of Interpretation. Doing qualitative research.

[29] Feldman SR, Krueger GG (2005). Psoriasis assessment tools in clinical trials. Ann Rheum Dis.

[30] Thomsen SF, Skov L, Dodge R, Hedegaard MS, Kjellberg J (2019). Socioeconomic Costs and Health Inequalities from Psoriasis: A Cohort Study. Dermatology (Basel, Switzerland).

[31] Naldi L, Addis A, Chimenti S, Giannetti A, Picardo M, Tomino C, Maccarone M, Chatenoud L, Bertuccio P, Caggese E (2008). Impact of body mass index and obesity on clinical response to systemic treatment for psoriasis. Evidence from the Psocare project. Dermatology (Basel, Switzerland).

[32] Hojgaard P, Glintborg B, Hetland ML, Hansen TH, Lage-Hansen PR, Petersen MH, Holland-Fischer M, Nilsson C, Loft AG, Andersen BN (2015). Association between tobacco smoking and response to tumour necrosis factor alpha inhibitor treatment in psoriatic arthritis: results from the DANBIO registry. Ann Rheum Dis.

[33] Glintborg B, Hojgaard P, Lund Hetland M, Steen Krogh N, Kollerup G, Jensen J, Chrysidis S, Jensen Hansen IM, Holland-Fischer M, Hojland Hansen T (2016). Impact of tobacco smoking on response to tumour necrosis factor-alpha inhibitor treatment in patients with ankylosing spondylitis: results from the Danish nationwide DANBIO registry. Rheumatology (Oxford, England).

[34] Schmidt M, Schmidt SAJ, Adelborg K, Sundbøll J, Laugesen K, Ehrenstein V, Sørensen HT (2019). The Danish health care system and epidemiological research: from health care contacts to database records. Clin Epidemiol.

[35] Henriksen DP, Rasmussen L, Hansen MR, Hallas J, Pottegård A (2015). Comparison of the five Danish regions regarding demographic characteristics, healthcare utilization, and medication use--a descriptive cross-sectional study. PLoS One.

[36] Kristensen LE, Jorgensen TS, Christensen R, Gudbergsen H, Dreyer L, Ballegaard C, Jacobsson LT, Strand V, Mease PJ, Kjellberg J. Societal costs and patients' experience of health inequities before and after diagnosis of psoriatic arthritis: a Danish cohort study. Ann Rheum Dis. 2017. 10.1136/annrheumdis-2016-210579. 10.1136/annrheumdis-2016-210579 28137915

[37] Menter A, Gottlieb A, Feldman SR, Van Voorhees AS, Leonardi CL, Gordon KB, Lebwohl M, Koo JY, Elmets CA, Korman NJ (2008). Guidelines of care for the management of psoriasis and psoriatic arthritis: section 1. Overview of psoriasis and guidelines of care for the treatment of psoriasis with biologics. J Am Acad Dermatol.

